# Medial open-wedge high tibial osteotomy alters sagittal tibial tubercle–trochlear groove distance

**DOI:** 10.1007/s00402-026-06205-7

**Published:** 2026-03-13

**Authors:** Sebastian Schmidt, Chilan Bou Ghosson Leite, Domenico Franco, Nicole Krabb, Sascha Gravius, Cale Andrew Jacobs, Christian Lattermann

**Affiliations:** 1 University Medical Centre Mannheim, Mannheim, Germany; 2 Brigham and Women’s Hospital, Boston, USA; 3 Landeskrankenhaus Feldkirch, Feldkirch, Austria

**Keywords:** MOWHTO, sTTTG, Joint loading, Tibial tubercle anteriorization

## Abstract

**Purpose:**

Medial open-wedge high tibial osteotomy (MOWHTO) is widely used to treat varus knee osteoarthritis, but its impact on patellofemoral biomechanics remains incompletely understood. In particular, the sagittal tibial tubercle–trochlear groove (sTT-TG) distance, a novel parameter linked to patellofemoral contact pressure, has not been evaluated in this context. This study aimed to assess changes in sTT-TG following MOWHTO and identify anatomical predictors of its postoperative magnitude.

**Methods:**

In this retrospective study, 34 knees from 33 patients (mean age 36.6 ± 9.5 years, mean BMI 26.2 ± 4.3 kg/m²) undergoing ascending biplanar MOWHTO with pre- and postoperative MRI and radiographs were analyzed. The sTT-TG, Caton–Deschamps Index (CDI), posterior tibial slope (PTS), and tibiofemoral rotation angle (TFRA) were measured. Correlation and multivariable regression analyses were performed to identify predictors of postoperative sTT-TG.

**Results:**

MOWHTO significantly decreased the sTT-TG distance from 6.25 ± 5.34 mm to 3.74 ± 6.81 mm (*p* = .009), indicating anteriorization of the tibial tubercle. Patellar height (CDI) decreased from 1.14 ± 0.20 to 0.99 ± 0.15 (*p* < .001), and TFRA was reduced from 4.74 ± 5.54° to 2.62 ± 5.50° (*p* = .017). Multivariable regression identified preoperative sTT-TG, postoperative medial PTS, and CDI as independent predictors of postoperative sTT-TG (adjusted R² = 0.697). A steeper medial PTS and lower patellar height were associated with greater tibial tubercle anteriorization.

**Conclusion:**

MOWHTO resulted in a significant anteriorization of the tibial tubercle in the sagittal plane. Postoperative sTT-TG is strongly influenced by tibial slope and patellar height, emphasizing the need to account for these factors during surgical planning, particularly in patients with patellofemoral cartilage changes. Future biomechanical studies should explore the clinical relevance of these changes on joint loading.

## Introduction

 Medial open-wedge high tibial osteotomy (MOWHTO) is a well-established surgical technique for the treatment of moderate unicompartmental medial knee osteoarthritis, particularly in young and patients with varus malalignment [1]. In addition to offloading the medial tibiofemoral compartment, MOWHTO is utilized in cases of focal medial femoral condyle cartilage defects to restore joint congruity and reduce focal loading [2]. While the procedure is primarily intended to address tibiofemoral pathology, growing evidence suggests that MOWHTO can induce secondary alterations in patellofemoral (PF) joint biomechanics by modifying radiographic alignment parameters [3, 4].

Two parameters, the Caton-Deschamps Index (CDI) and the tibial tubercle–trochlear groove (TT-TG) distance, frequently change following MOWHTO [3–6]. Specifically a reduction in patellar height has been frequently reported, which may elevate PF contact forces and predispose patients to anterior knee pain or secondary patellofemoral cartilage degeneration [5–7]. These changes are particularly relevant when managing existing patellofemoral pathology or when MOWHTO is considered for patients with combined tibiofemoral and patellofemoral disease.

In this context, a recently introduced imaging parameter, the sagittal TT-TG (sTT-TG) distance, has emerged as a potentially more comprehensive surrogate for evaluating patellofemoral joint loading [8–10]. Several studies have shown that patients with a more posterior tibial tubercle exhibit more frequent and severe patellofemoral cartilage lesions [9–11].

Given that posterior tibial tubercle positioning can contribute to increased patellofemoral contact pressure, sTT-TG may serve as a sensitive indicator for alterations in patellofemoral mechanics following MOWHTO.

Despite its potential relevance, the impact of MOWHTO on sTT-TG distance remains poorly understood, as do the factors influencing its postoperative magnitude. A better understanding of these relationships could help refine preoperative planning and improve patient selection, especially for those at risk for patellofemoral disorders.

Therefore, the purpose of this study was to assess changes in the sTT-TG distance following MOWHTO and to identify anatomical or radiographic factors associated with these alterations. It was hypothesized that MOWHTO significantly alters the sTT-TG distance and that changes in patellar height and joint line orientation may influence the sTT-TG.

## Methods

The study received approval from the institutional review board (2020P003747).

### Patient selection

The indication for MOWHTO was symptomatic medial compartment overload associated varus malalignment. This included patients with early medial osteoarthritis as well as those with focal medial femoral condyle cartilage defects and/or meniscal deficiency requiring concomitant cartilage or meniscal restoration.

Medical records were retrospectively reviewed to identify patients aged 18 years or older who underwent ascending biplanar MOWHTO between 2017 and 2023. To ensure reliable assessment of alignment correction and patellofemoral parameters, only patients with complete preoperative and postoperative knee magnetic resonance imaging (MRI) and standardized anteroposterior long-leg and true lateral knee radiographs were included. Patients were excluded if they had undergone prior bony realignment procedures around the knee, if a concomitant femoral or tibial tubercle osteotomy was performed, or if ligamentous instability was present at the time of preoperative or follow-up imaging. Ligamentous instability was defined as clinically apparent instability and/or ligamentous abnormalities detected on MRI.

Demographic parameters, including age, body mass index (BMI), affected side, and concomitant procedures, were recorded (Table 1).

### Surgical technique

All procedures were performed using a standardized surgical technique based on preoperative planning with the Miniaci method [12]. The correction aimed to shift the weight-bearing axis to a point between 50% and 70% across the tibial plateau, depending on the degree of lateralization desired by the operating surgeon. Intraoperatively, the staggered osteotome technique was employed to create the planned biplanar medial opening wedge under fluoroscopic guidance. Fixation was achieved using a medial locking plate. When indicated, concomitant procedures—such as osteochondral allograft (OCA) transplantation or matrix-associated autologous chondrocyte implantation (MACI)—were performed during the same surgical session. Postoperative rehabilitation included touch-down weight bearing with crutches for the first four weeks. Knee flexion was limited to 90° during the initial three weeks and gradually increased thereafter. Stationary cycling was initiated after six weeks, while running was permitted after a minimum of four months, contigent upon radiographic evidence of bone healing was confirmed. High-impact activities and pivoting sports were generally restricted until at least six months postoperatively.

### Imaging evaluation

For patients with multiple MRI scans and radiographs, the preoperative imaging closest to the date of the MOWHTO was selected for analysis. MRI examinations performed on both 1.5-T and 3.0-T scanners were included. All measurements were conducted using standardized angle and distance tools within the institution’s picture archiving and communication system (PACS).

Joint line orientation was assessed as a composite concept encompassing both coronal and sagittal joint line geometry. Coronal joint line orientation was represented by the medial mechanical proximal tibial angle (mMPTA), while sagittal joint line orientation was characterized by posterior tibial slope (PTS) and joint line elevation, approximated by the tibial tubercle–joint line (TT–JL) distance.

### Sagittal TT-TG distance

The sTT–TG distance was measured on axial MRI as previously described [8, 10, 13]. The trochlear reference point was defined as the deepest point of the trochlear groove based on the articular cartilage surface on the axial slice demonstrating the Roman arch. A reference line perpendicular to the posterior femoral condylar axis was drawn through this point. The most prominent point of the tibial tubercle was then identified, and its anteroposterior offset relative to the trochlear reference line was measured.

### TT-TG distance

The TT–TG distance was defined as the mediolateral offset between the deepest point of the trochlear groove—identified on the axial MRI slice showing the Roman arch—and the center of the tibial tubercle, measured along lines drawn perpendicular to the posterior femoral condylar axis [14].

### Tibiofemoral rotation angle

**Fig. 1:**
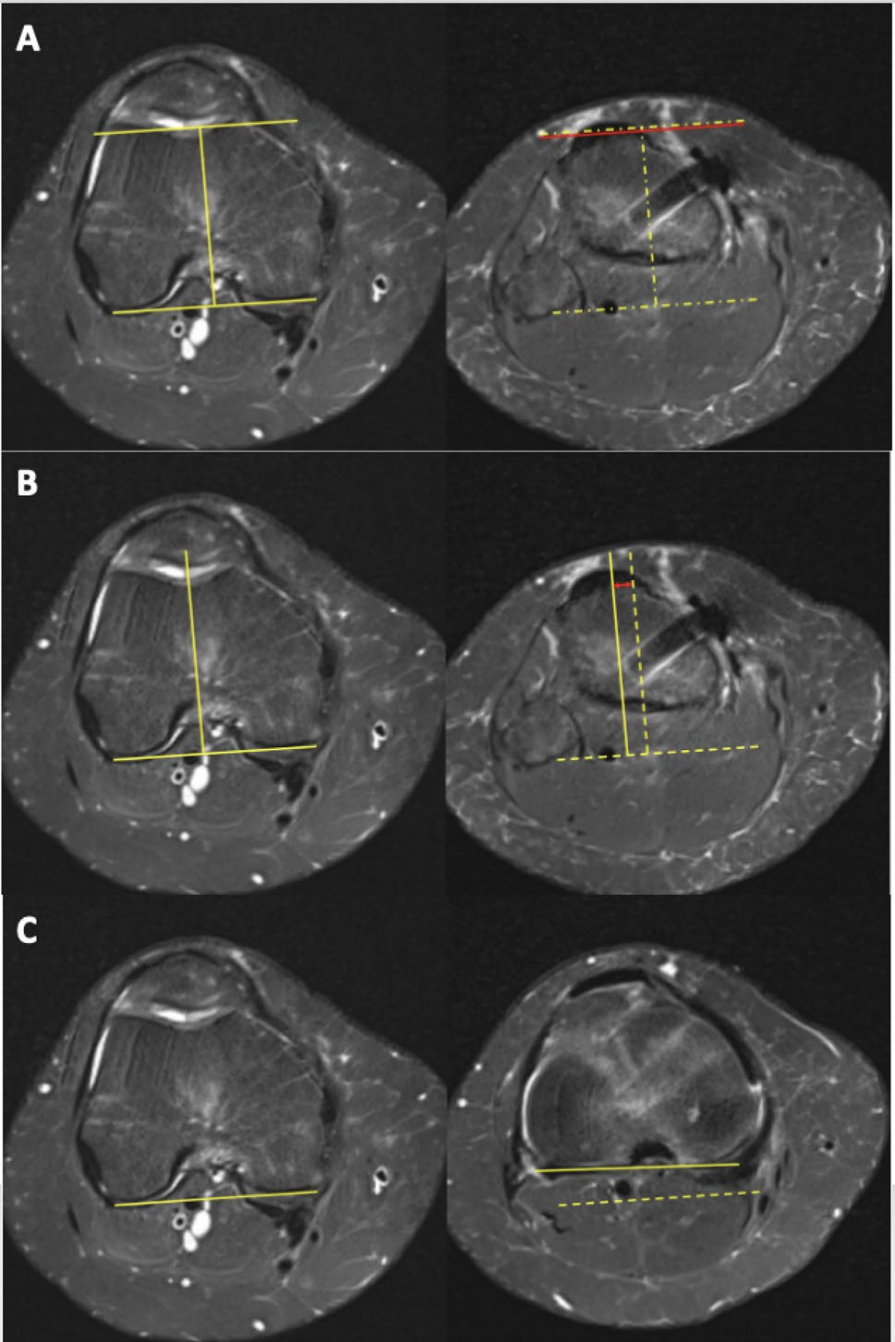
MRI-based measurement techniques. **A** Sagittal tibial tubercle–trochlear groove (sTT–TG) distance: The deepest point of the trochlear groove was identified on the axial slice showing the Roman arch, and a line perpendicular to the posterior femoral condylar axis was drawn (left panel, yellow lines). The most prominent point of the tibial tubercle was located, and its anteroposterior distance relative to the trochlear groove was measured (right panel, vertical red line to the yellow line). A positive value indicated a posteriorly positioned tibial tubercle. **B** Tibial tubercle–trochlear groove (TT–TG) distance: The mediolateral distance (red double arrow) between the trochlear groove and the tibial tubercle was measured on axial images, using perpendicular lines referenced to the posterior condylar axis (yellow lines). **C** Tibiofemoral rotation angle (TFRA): Defined as the angle between the posterior femoral condylar axis (yellow line, left) and the posterior tibial axis just distal to the tibial plateau (yellow line, right). A positive angle indicated internal tibial rotation relative to the femur

The tibiofemoral rotation angle (TFRA) at the level of the knee joint was assessed on axial MRI in accordance with previously described methods [15]. TFRA was defined as the angle between the posterior femoral condylar axis—measured at the level of the deepest point of the trochlear groove—and the posterior tibial axis—measured just distal to the tibial plateau articular surface. A positive TFRA value indicated internal rotation of the tibia relative to the femur (Fig. 1).

### Posterior tibial slope

The posterior tibial slope (PTS) was measured according to established protocols [15, 16]. On midsagittal MRI, the tibial longitudinal axis was determined using two circles: a proximal circle fitted to the subchondral cortices of the tibial plateau and a distal circle aligned with the anterior and posterior metaphyseal cortices, with its center constrained to the circumference of the proximal circle. A line connecting the centers of both circles defined the tibial axis. Medial and lateral PTS were then measured on their respective mid-sagittal slices as the angle between a line perpendicular to the tibial axis and a tangent to the articular surface of each tibial plateau.

### Knee flexion assessment

**Fig. 2:**
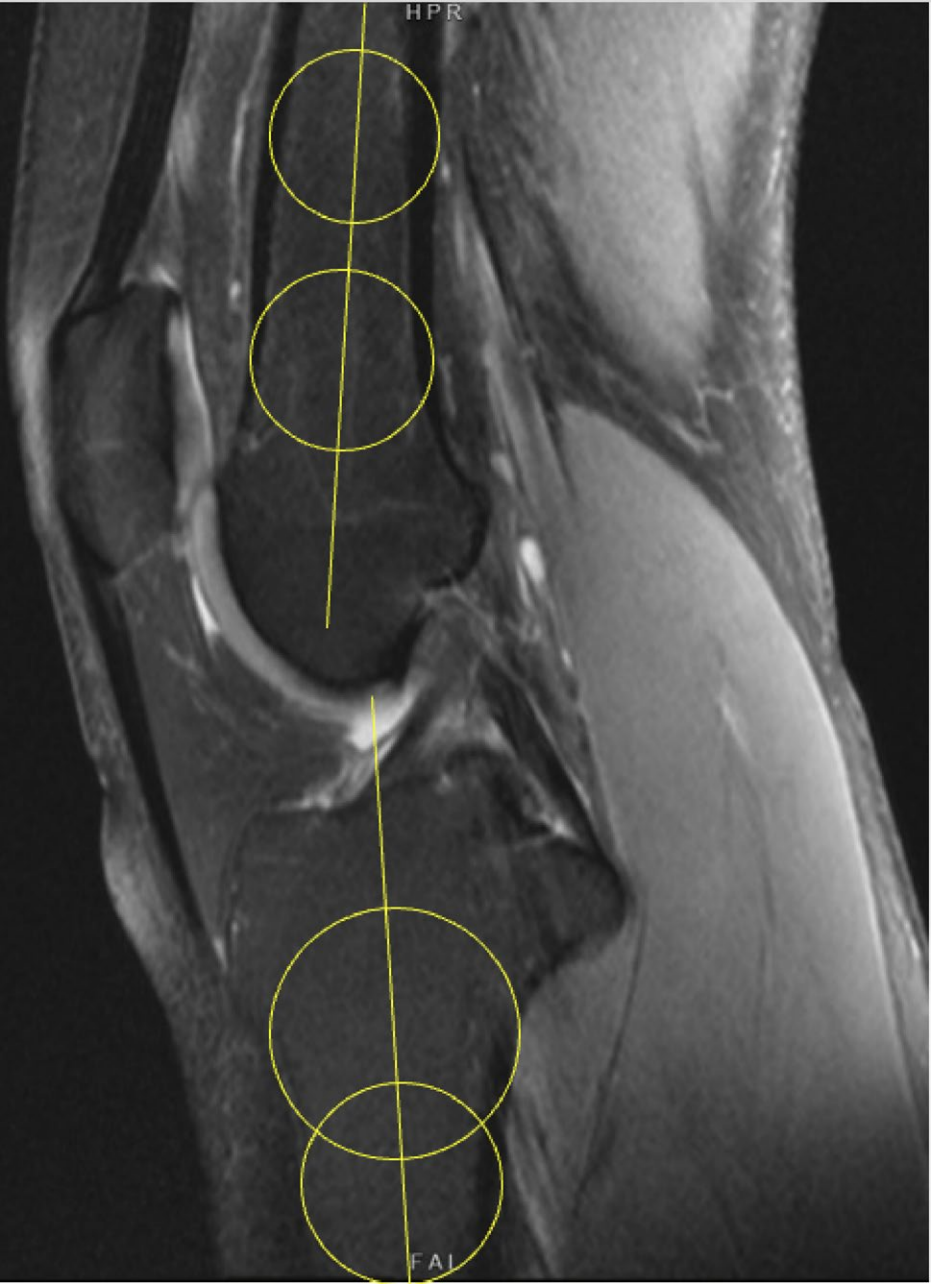
MRI-based measurement of the knee flexion angle

As an additional parameter, the knee flexion angle on MRI was determined using the mid-sagittal slice by measuring the angle between the longitudinal axes of the femur and tibia (Fig. 2).

### Plain radiograph assessments

**Fig. 3:**
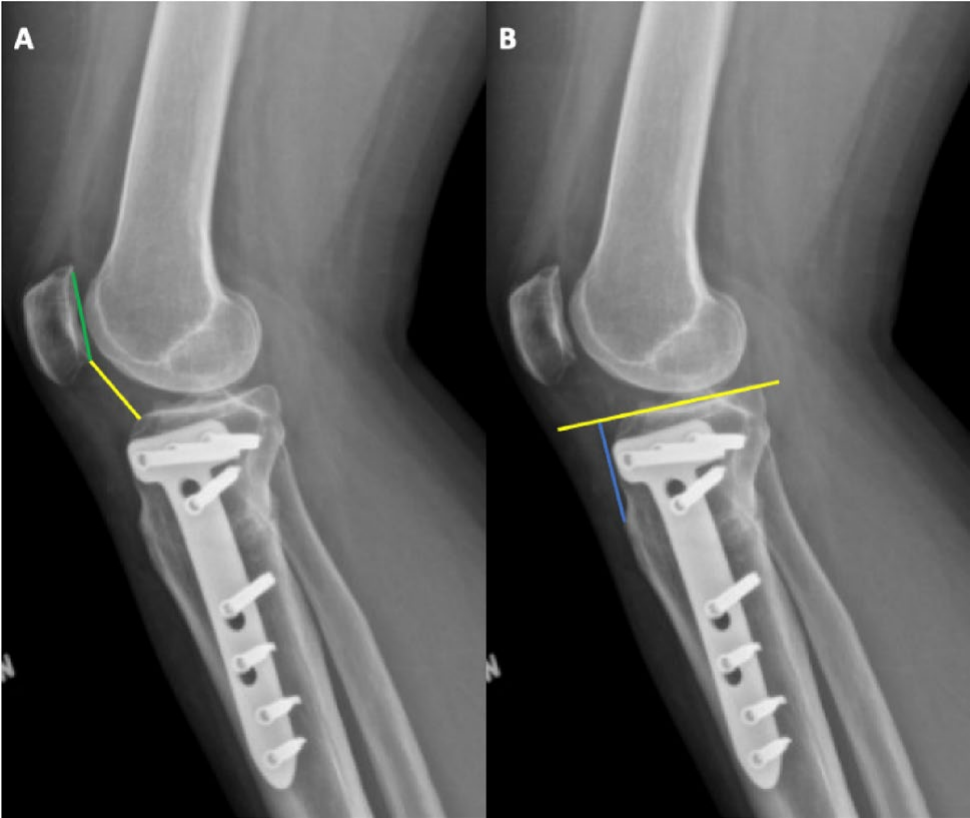
Radiographic measurement of patellar height and joint line position. **A** Caton–Deschamps Index (CDI): Defined as the ratio between the distance from the inferior pole of the patella to the anterosuperior aspect of the tibial plateau (yellow line) and the length of the patellar articular surface (green line). **B** Tibial tubercle–joint line (TT–JL) distance: Measured as the perpendicular distance (blue line) from a line along the tibial plateau surface (yellow line) to the most prominent point of the tibial tubercle

Plain radiographs were assessed using standardized AP long-leg and true lateral views of the knee. On the AP long-leg radiograph, the hip–knee–ankle (HKA) angle and the medial mechanical proximal tibial angle (mMPTA) were measured to evaluate coronal alignment and the degree of correction. On the true lateral radiograph, patellar height was assessed using CDI, defined as the ratio between the distance from the inferior pole of the patella to the anterior edge of the tibial plateau and the length of the patellar articular surface [17]. As an exploratory measurement, the tibial tubercle–joint line (TT–JL) distance was calculated as an approximation of joint line elevation (Fig. 3). For this purpose, a line was drawn along the tibial plateau, and a second line was placed perpendicular to it, extending from the plateau to the most prominent point of the tibial tubercle; the resulting perpendicular distance was recorded. Furthermore, on the postoperative AP radiograph, the medial cortical gap, the distance between the proximal and distal tibial cortices at the osteotomy site, was measured to quantify the extent of wedge opening.

### Statistical analysis

Descriptive statistics are presented as mean ± standard deviation (SD) for continuous variables and as absolute frequencies with corresponding percentages for categorical variables. Data distribution and normality were assessed using histogram inspection and the Shapiro–Wilk test. Comparisons of continuous variables were conducted using either the paired *t* test or the Wilcoxon signed-rank test, as appropriate based on data distribution. Inter-rater and intra-rater reliability were evaluated using intraclass correlation coefficients (ICC) based on a two-way random-effects model with absolute agreement. For intra-rater reliability, measurements were repeated after a minimum interval of 8 weeks. An ICC ≥ 0.75 was considered good and an ICC ≥ 0.90 was considered excellent. Spearman correlation analysis was performed to assess associations between postoperative sTT-TG and other preoperative and postoperative parameters. All parameters that showed a significant correlation with sTT-TG in the univariable analysis were subsequently included in a multivariable linear regression model to identify independent predictors. A post hoc power analysis was conducted based on the observed effect size (adjusted R² = 0.697). This analysis revealed that a minimum of 14 patients was sufficient to achieve 80% statistical power at an alpha level of 0.05. Statistical analysis was performed using R version 4.2.3, and a *p*-value < 0.05 was considered statistically significant.

## Results

A total of 34 knees from 33 patients were included in this study. Sixteen patients underwent at least one concomitant procedure. One patient underwent two concomitant procedures, meniscal allograft transplantation and osteochondral allograft transplantation, resulting in a total of 17 procedures, as detailed in Table 1.

**Table 1:**
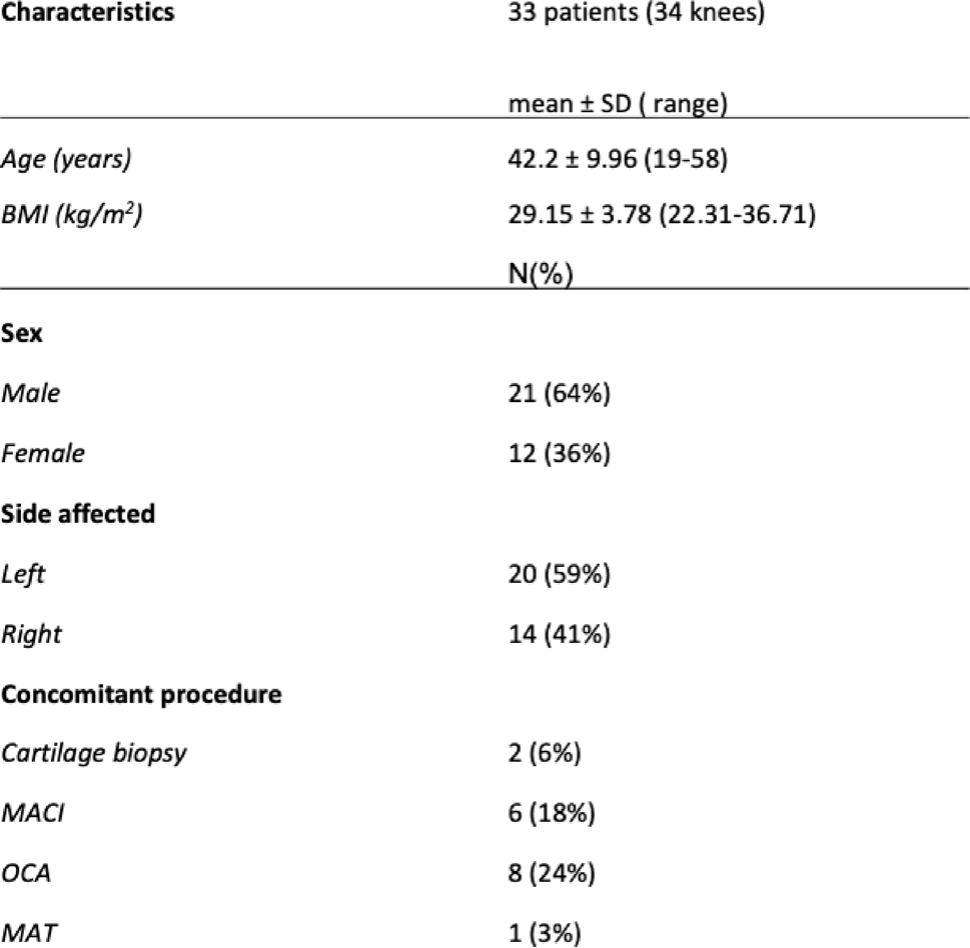
Patient demographics and concomitant procedures

*BMI* Body mass index, *MACI* Matrix-associated autologous chondrocyte implantation, *OCA* Osteochondral allograft transplantation, *MAT* Meniscal allograft transplantation

**Table 2:**
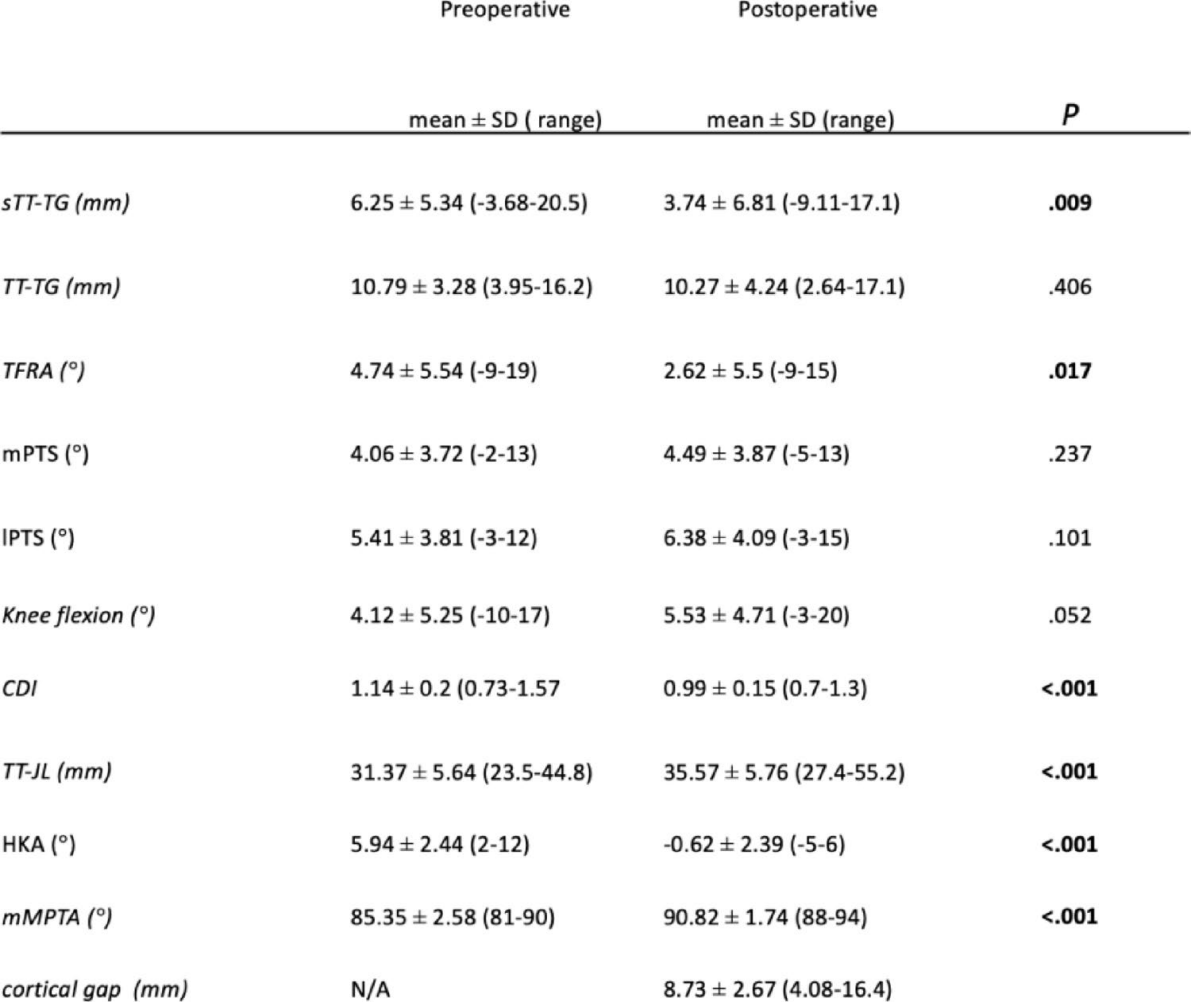
Radiographic and MRI-based changes following medial open-wedge high tibial osteotomy (MOWHTO)

*sTT-TG* Sagittal tibial tubercle–trochlear groove distance, *TT-TG* Tibial tubercle–trochlear groove distance (axial), *TFRA* Tibiofemoral rotation angle, *mPTS* Medial posterior tibial slope, *lPTS* Lateral posterior tibial slope, *CDI* Caton–deschamps Index, *TT-JL* Tibial tubercle–joint line distance, *HKA* Hip–knee–ankle angle, *mMPTA* Medial mechanical proximal tibial angle

Bold P value indicates statistical significance (*P* <.05)

A significant correction of coronal alignment was achieved, with the hip–knee–ankle angle changing from 5.94 ± 2.44° preoperatively to − 0.62 ± 2.39° postoperatively (*p* <.001) and the medial mechanical proximal tibial angle increasing from 85.35 ± 2.58° to 90.82 ± 1.74° (*p* <.001). The mean medial cortical gap was 8.73 ± 2.67 mm. The sagittal tibial tubercle–trochlear groove distance decreased from 6.25 ± 5.34 mm to 3.74 ± 6.81 mm (*p* =.009), the tibiofemoral rotation angle decreased from 4.74 ± 5.54° to 2.62 ± 5.50° (*p* =.017), and the Caton–Deschamps Index decreased from 1.14 ± 0.20 to 0.99 ± 0.15 (*p* <.001). No other measured parameters showed statistically significant postoperative changes (Table 2).

Interrater reliability ranged from good to excellent (ICC 0.78–0.97) across all measured parameters, including the hip–knee–ankle angle, medial proximal tibial angle, anatomic–mechanical femoral angle, Caton–Deschamps index, joint line–to–tibial tuberosity distance, MRI flexion angle, medial and lateral posterior tibial slope, tibiofemoral rotation angle, tibial tuberosity–trochlear groove distance, and sagittal tibial tuberosity–trochlear groove distance.

Intrarater reliability was good to excellent for all measurements (ICC 0.87–0.96).

Spearman correlation analysis revealed that higher postoperative sTT-TG values were significantly associated with greater preoperative sTT-TG (ρ = 0.652, *p* <.001), lower mPTS both pre- (ρ = −0.533, *p* =.001) and postoperatively (ρ = −0.615, *p* <.001), as well as with a lower lPTS (ρ = −0.457, *p* =.006), a higher CDI preoperatively (ρ = 0.357, *p* =.035), and increased postoperative knee flexion angle (ρ = 0.384, *p* =.023). Additionally, preoperative TFRA was negatively correlated with postoperative sTTTG (ρ = −0.382, *p* =.024), indicating that less external tibial rotation is associated with anterior positioning of the tibial tubercle.

Multivariable linear regression identified several independent predictors of postoperative sTT-TG (adjusted R² = 0.697, *p* <.001). The strongest association was found for preoperative sTT-TG (β = 0.66, 95% CI: 0.37 to 0.95, *p* <.001), indicating that higher preoperative values are associated with persistently anterior tubercle positions after surgery. A steeper postoperative mPTS was also significantly associated with increased postoperative anteriorization (β = −0.70, 95% CI: −1.11 to − 0.28, *p* =.002).

Postoperative patellar height showed an inverse relationship with postoperative sTT-TG (β = −11.13, 95% CI: −22.18 to − 0.02, *p* =.049), suggesting that a lower patella position may be linked to anterior tibial tubercle displacement. Therefore a 1.44° steeper mPTS or an increase in the CDI by 0.1 was associated with a 1 mm decrease in the sTT-TG, indicating a more anterior position of the tibial tubercle. Other predictors were not significantly associated (Table 3).

**Table 3:**
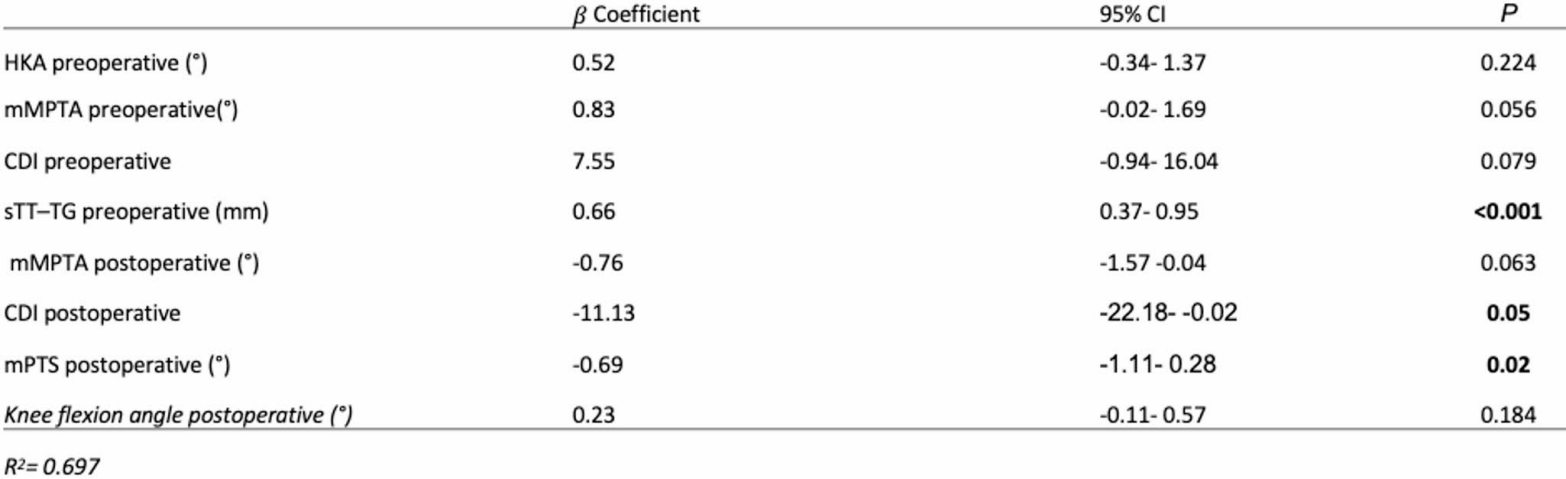
Multivariable linear regression analysis identifying factors associated with postoperative sTT-TG distance

Regression coefficients (β), 95% confidence intervals (CI), and p values are shown for all variables included in the model

The overall model demonstrated good explanatory power (adjusted R² = 0.697)

*HKA* Hip–knee–ankle angle, *mMPTA* Medial mechanical proximal tibial angle, *CDI* Caton–deschamps index, *sTT–TG* Sagittal tibial tubercle–trochlear groove distance, *mPTS* Medial posterior tibial slope

## Discussion

The most important finding of this study is that MOWHTO significantly reduces the sTT-TG distance, indicating a postoperative anteriorization of the tibial tubercle relative to the trochlear groove. This novel observation underscores that MOWHTO not only induces coronal and sagittal realignment but also substantial changes of the patellofemoral biomechanics in the sagittal plane and regarding TFRA also in the axial plane. Given the growing interest in using sTT-TG as a surrogate for PF loading, these results are of high clinical relevance and may inform preoperative planning in patients at risk of PF joint overload.

A significant and reliable correction of coronal alignment was achieved in this cohort, as evidenced by the marked changes in HKA and mMPTA. These results confirm the accuracy and reproducibility of the surgical technique and are consistent with previous studies [1, 18, 19].

Several studies have shown that patients with a more posterior tibial tubercle exhibit more frequent and severe patellofemoral cartilage lesions [9–11]. MOWHTO reduced sTT-TG distance significantly in this study, which could theoretically protect cartilage by decreasing PF loading. This is in line with a study by Sim et al., which evaluated PF compartimental changes according to SPECT/CT analysis after MOWHTO [20]. They found that alignment correction by ascending MOWHTO resulted in PF compartment offloading.

The observed decrease in sTT–TG after MOHTO is most likely attributable to combined sagittal and axial alignment changes. A primary mechanism is alteration of posterior tibial slope, as an increased slope effectively promotes anterior tibial translation relative to the femur, thereby modifying the relative anteroposterior relationship between the tibial tubercle and the trochlear groove. This interpretation is supported by our finding that postoperative medial posterior tibial slope independently predicted postoperative sTT–TG and is consistent with prior biomechanical and clinical observations following MOWHTO [3, 4]. In addition, several studies have demonstrated that MOWHTO can induce rotational changes of the distal tibia, depending on osteotomy plane orientation and wedge opening mechanics [21, 22]. Such rotational effects provide a coherent explanation for postoperative changes in axial parameters, including TT–TG changes reported previously [4] and the tibiofemoral rotation angle changes observed in our cohort. Together, these findings suggest that MOWHTO can influence extensor mechanism alignment in multiple planes, emphasizing the importance of slope preservation and precise osteotomy execution during surgical planning.

The decrease in patellar height, especially following ascending MOWHTO is a well-documented and consistent finding across the literature, regardless of the specific measurement method used [6]. In line with these findings, our study also demonstrated a significant decrease in patellar height postoperatively. The amount of correction is a significant factor affecting patella height and with increased correction in the coronal plane the likelihood of influencing and decreasing the patella height increases in the literature [6].

Despite the consistent decrease in patellar height after MOWHTO, it remains unclear whether this change contributes to the development of patellofemoral osteoarthritis (PFOA), as several studies found no significant differences in patellar height between groups with and without PFOA progression [5, 6, 23–25]. So far, systematic reviews suggest that PFOA progression after open-wedge high tibial osteotomy is more closely associated with the degree of alignment correction or the severity of preoperative varus malalignment [5, 6]. Therefore, it is possible that the PF joint was nonetheless unloaded due to the concurrent anteriorization of the tibial tubercle, although patellar height decreased. Notably, patellar height emerged as an independent predictor of postoperative sTT-TG in our regression analysis, suggesting that patella baja may be directly linked to posterior displacement of the tibial tubercle in the sagittal plane. This highlights the need to consider patellar height not only as an isolated outcome parameter but also as a biomechanically relevant factor influencing sagittal patellofemoral alignment. Future studies should investigate the combined effects of changes in sTT-TG and patellar height on patellofemoral contact pressure after MOWHTO using biomechanical testing or finite element modeling to clarify their clinical implications.

Furthermore, the clinical relevance of PFOA progression following MOWHTO remains uncertain [5, 6]. While some studies have reported that radiographic progression of PFOA does not significantly impact clinical outcomes in the mid- to long-term, two recent systematic reviews concluded that current evidence is insufficient to draw definitive conclusions [5, 6, 18, 19, 26]. This is largely due to the heterogeneity of outcome measures used across studies and the relatively small sample sizes, which limit the generalizability of findings. As a result, the true clinical and functional implications of PFOA progression and patellar height changes after MOWHTO remain inconclusive and warrant further investigation in larger, well-designed trials [6].

In addition to the observed anatomical predictors, tibiofemoral rotation changed significantly following MOWHTO, with a postoperative decrease in TFRA indicating a more externally rotated tibial position relative to the femur. The association between MOWHTO and rotational alignment has only recently come under investigation [3, 4, 27]. Although no prior studies have specifically analyzed TFRA, several have evaluated TT-TG as a surrogate marker of axial alignment [3, 4, 28]. Unlike the findings in this study, these studies reported significant postoperative increases in TT-TG after MOWHTO. The exact mechanism behind these changes remains unclear. Some authors propose that an osteotomy plane not perfectly perpendicular to the mechanical axis induces unintentional rotation during wedge opening [21]. Others have implicated alterations in the lPTS [22, 29]. However, this study found no significant postoperative change in lPTS, arguing against this explanation.

Although TFRA was not an independent predictor of postoperative sTT-TG in the multivariable model, it remains clinically relevant. Recent data by Leite et al. demonstrated that a TFRA ≥ 4.5° significantly increases the risk of ACL graft failure, with affected patients showing a higher failure rate and reduced graft survival [15]. In the present cohort, MOWHTO resulted in a reduction of TFRA below this threshold in most patients, suggesting that the procedure may favorably influence axial alignment. This observation is particularly relevant in patients undergoing MOWHTO in conjunction with, or prior to, ACL reconstruction or revision.

Importantly, these results advocate for an individualized approach in surgical planning. In patients with concomitant PF symptoms or borderline anatomical risk factors (e.g. preoperative sTT-TG higher than 10 mm [11]), strategies to preserve tibial slope or maintain patellar height, such as minimizing joint line elevation, selecting lower opening wedge sizes or performing descending MOWHTO could mitigate some of the adverse patellofemoral changes [30].

### Limitations

Several limitations must be acknowledged. First, although the sample size was relatively small, this was a consequence of strict inclusion and exclusion criteria designed to ensure a homogeneous and reliable patient cohort. In particular, patients with any evidence of ligamentous instability or prior bony realignment procedures were excluded to eliminate confounding effects on tibial tubercle positioning. Only patients who underwent surgery by high-volume knee surgeons using a standardized ascending biplanar osteotomy technique were included to ensure procedural consistency and high technical quality. Although minor inter-surgeon variability in osteotomy plane orientation cannot be completely excluded, this approach improves internal validity. However, the results may be less generalizable to lower-volume centers or broader surgical populations. Second, MRI-based measurements such as the sTT-TG are inherently dependent on the degree of knee flexion at the time of imaging. Although we included the MRI flexion angle as a covariate in the regression model to account for this variability, some degree of residual confounding due to variable imaging conditions cannot be entirely excluded. Third, while the observed changes in sTT-TG and their anatomical predictors are biomechanically plausible and supported by prior literature, this study did not include clinical or functional outcome data. Therefore, the clinical significance of postoperative sTT-TG alterations remains speculative. Future studies should seek to correlate changes in sTT-TG with patient-reported outcomes, anterior knee pain, and measures of patellofemoral joint function or OA to better understand the clinical impact of these biomechanical changes.

## Conclusion

MOWHTO resulted in a significant anteriorization of the tibial tubercle in the sagittal plane. Postoperative sTT-TG is strongly influenced by tibial slope and patellar height, emphasizing the need to account for these factors during surgical planning, particularly in patients with patellofemoral cartilage changes. Future biomechanical studies should explore the clinical relevance of these changes on joint loading.

## Data Availability

No datasets were generated or analysed during the current study.
